# PD31 Counting Oncology Patients In The Brazilian Public Health System: A Comprehensive New Methodology

**DOI:** 10.1017/S0266462325101657

**Published:** 2025-12-29

**Authors:** Patricia Klarmann Ziegelmann, Cintia Sayuri Kurokawa La Scala, Nelson Francisco Correa-Netto

## Abstract

**Introduction:**

In Brazil, the databases of the informatics department of the Brazilian Unified Health System (DATASUS) are key sources of oncology real-world data (RWD). Despite challenges like data quality, transparent methods ensure robust real-world evidence for cancer care. This study proposed a reproducible and open-sourced methodology to count cancer cases in the Unified Health System (SUS).

**Methods:**

The methodology counts cancer cases treated in the SUS using claims data available from DATASUS. The unique patient counting system employs patient ID, with deduplication for patients with more than one ID, using a key based on sex, ZIP code, International Classification of Diseases, 10th Revision (ICD-10) codes, diagnosis date, and calculated birth year. This new methodology, which includes data download, was implemented in an R software package. Data from DATASUS outpatient records (chemotherapy, immunotherapy, or radiotherapy) for patients aged 18 to 99 years with pancreatic cancer (ICD-10 code C25) diagnosed between 2008 and 2022 were used to validate the method.

**Results:**

A total of 254,240 outpatient claim records were analyzed: counting 31,425 unique patients by the proposed methodology and 1.3 percent of records where the patient ID represented duplication. Patients had a mean age of 60.9 years, 50.2 percent were female, and 82.8 percent were aged 50 years or older. Regarding the cancer stage, 3.5 percent had in situ, whereas 3.4 percent, 9.3 percent, 17.6 percent, and 66.2 percent had stages I, II, III, and IV, respectively.

**Conclusions:**

The study highlighted how a database created for administrative purposes can be used to describe cancer care. The open-source availability of the methodology (via an R package) ensures transparency and reproducibility in RWD analysis.

